# Insight into the Molecular and Functional Diversity of Cnidarian Neuropeptides

**DOI:** 10.3390/ijms16022610

**Published:** 2015-01-23

**Authors:** Toshio Takahashi, Noriyo Takeda

**Affiliations:** 1 Suntory Foundation for Life Sciences, Bioorganic Research Institute, Osaka 618-8503, Japan; 2 Research Center for Marine Biology, Asamushi, Graduate School of Life Sciences, Tohoku University, Aomori 039-3501, Japan; E-Mail: ntakeda@m.tohoku.ac.jp

**Keywords:** cnidaria, hydra, jellyfish, coral, neuropeptide

## Abstract

Cnidarians are the most primitive animals to possess a nervous system. This phylum is composed of the classes Scyphozoa (jellyfish), Cubozoa (box jellyfish), and Hydrozoa (e.g., *Hydra*, *Hydractinia*), which make up the subphylum Medusozoa, as well as the class Anthozoa (sea anemones and corals). Neuropeptides have an early evolutionary origin and are already abundant in cnidarians. For example, from the cnidarian *Hydra*, a key model system for studying the peptides involved in developmental and physiological processes, we identified a wide variety of novel neuropeptides from *Hydra magnipapillata* (the *Hydra* Peptide Project). Most of these peptides act directly on muscle cells and induce contraction and relaxation. Some peptides are involved in cell differentiation and morphogenesis. In this review, we describe FMRFamide-like peptides (FLPs), GLWamide-family peptides, and the neuropeptide Hym-355; FPQSFLPRGamide. Several hundred FLPs have been isolated from invertebrate animals such as cnidarians. GLWamide-family peptides function as signaling molecules in muscle contraction, metamorphosis, and settlement in cnidarians. Hym-355; FPQSFLPRGamide enhances neuronal differentiation in *Hydra*. Recently, GLWamide-family peptides and Hym-355; FPQSFLPRGamide were shown to trigger oocyte maturation and subsequent spawning in the hydrozoan jellyfish *Cytaeis uchidae*. These findings suggest the importance of these neuropeptides in both developmental and physiological processes.

## 1. Introduction

Neurotransmission is the process by which signaling molecules called neurotransmitters, including peptides, are released by a neuron and bind to and activate the receptors of another neuron. Neuropeptides include small peptides and peptide hormones derived from nerve cells and range from as short as three amino acids (e.g., thyrotropin-releasing hormone) [1] to as long as 70 or more (e.g., eclosion hormone) [2]. Neuropeptide receptors are primarily members of the large family of G protein-coupled receptors (GPCRs), although notable exceptions are found. Some neuropeptides directly gate ion channels [3], whereas insulin, which is a neuropeptide in some invertebrates [4], signals through its traditional tyrosine kinase insulin receptor. Finally, neurons secrete a multitude of other proteinaceous factors (e.g., growth factors) that signal through diverse receptor types. We will restrict this review to a discussion of three types of neuropeptides found in cnidarians. Readers interested in GPCRs may refer to a recent excellent review in cnidarians [5].

Cnidarians have a diffuse nervous system including a nerve net in which the sensory and ganglionic neurons and their processes are interspersed among the epithelial cells of both layers. Cnidarian nervous systems are strongly peptidergic, not solely peptidergic [6]. Classical neurotransmitters such as the biogenic amines that have long been studied as major neurotransmitters in higher eukaryotes are also involved in cnidarian neurotransmission [7]. The prevailing view of the nervous system in the freshwater polyp *Hydra* (Hydrozoa) is that the neuronal network is simple and diffuse throughout the animal’s body. As to the hydrozoan medusae, marginal nerve rings and ganglion-like structures associated with sensory organs are observed. Using the model organism *Hydra*, which can be used to study not only cell biological and regenerative mechanisms but also physiological processes regulated by peptide signaling molecules, we developed a novel peptidomic approach to the isolation and identification of functional peptide signaling molecules for this organism (the *Hydra* Peptide Project) [8]. Over the course of this project, we identified a variety of neuropeptides. Most of these peptides are novel, but some peptides are homologous to peptide families that have previously been identified.

For example, GLWamide-family peptides (GLWamides), which were first isolated from the sea anemone *Anthopleura elegantissima* [9] and then from *Hydra magnipapillata* [8], induce the metamorphosis of *Hydractinia serrata* planula larvae into polyps. In *Hydra*, the peptides induce detachment of the bud from a parental polyp [8]. The neuropeptide Hym-355; FPQSFLPRGamide enhances neuronal differentiation by inducing multipotent interstitial stem cells to enter the neuronal differentiation pathway [10]. Grimmelikhuijzen and colleagues isolated four FMRFamide-like peptides (FLPs) from *Hydra* using a radioimmunoassay method [11]. We also identified the same peptides using high-throughput reverse-phase nano-flow matrix-assisted laser desorption/ionization mass spectrometry (LC-MALDI-MS/MS) [12]. LC-MALDI-MS/MS is a powerful tool that has been widely used in the study of biological systems [13,14]. Accumulating evidence suggests that *Hydra* peptides exist and function beyond cnidarian species.

In this review, we describe FLPs, GLWamide-family peptides, and Hym-355; FPQSFLPRGamide, which have diverse functions as neuropeptides in cnidarians, including two species of Hydrozoa (*Hydra*, *Hydractinia*, and jellyfish) and Anthozoa (coral). We also discuss the importance of the neuropeptides in the development and physiology of the cnidarians.

## 2. Cnidarian Neuropeptides

### 2.1. FLPs (FMRFamide-Like Peptides)

The peptide FMRFamide, which is composed of four amino acid residues with *C*-terminal amidation, was first isolated from the cerebral ganglion of the clam *Macrocallista nimbosa* [15,16]. To date, peptides sharing a similar sequence have been isolated from other mollusks and from members of most other phyla. These peptides are now divided into two groups according to the level of structural similarity compared with FMRFamide. One group is FMRFamide-related peptides (FaRPs), which contain *N*-terminal extensions of the *C*-terminal FMRFamide or FLRFamide core sequences [17]. The other group is FLPs, which include all peptides with the RFamide sequence only [18]. Thus, FLPs include FaRPs and all other RFamide peptides. An excellent review has examined FaRPs from invertebrate animals [19]. The main focus of this review will be on cnidarian FLPs.

The evolutionarily “ancient” nervous systems in cnidarians express a variety of FLPs (Table 1). Peptides with GRFamide at the *C*-terminus have been found in a scyphozoan [20], three hydrozoans [11,21,22,23,24,25], and an anthozoan [26], whereas peptides with TRFamide and/or RRFamide at the *C*-terminus have been sequenced from another anthozoan [27].

**Table 1:** FMRFamide-like peptides in Cnidaria.

Name	Peptide Sequence	Species	Reference
Antho-RFamide	pQGRFamide	*Anthopleura elegantissima*	[26]
Cyanea-RFamide I	pQWLRGRFamide	*Cyanea Lamarckii*	[20]
Cyanea-RFamide II	pQPLWSGRFamide		
Cyanea-RFamide III	GRFamide		
Pol-RFamide I	pQLLGGRFamide	*Polyorchis penicillatus*	[21]
Pol-RFamide II	pQWLKGRFamide		[22]
Hydra-RFamide I	pQWLGGRFamide	*Hydra magnipapillata*	[11]
Hydra-RFamide II	pQWFNGRFamide		
Hydra-RFamide III	KPHLRGRFamide		
Hydra-RFamide IV	HLRGRFamide		
Hydra-RFamide V	pQLMSGRFamide	*Hydra magnipapillata*	[23]
Hydra-RFamide VI	pQLMRGRFamide		
Hydra-RFamide VII	pQLLRGRFamide		
Hydra-RFamide VIII	KPHYRGRFamide		
Hydra-RFamide IX	HYRGRFamide		
Hydra-RFamide X	KPHLIGRFamide	*Hydra magnipapillata*	[24]
Hydra-RFamide XI	pQLMTGRFamide		
He-RFamide	pQWLKGRFamide	*Hydractinia echinata*	[25]
Nv-RFamide I	pQITRFamide	*Nematostella vectensis*	[27]
Nv-RFamide II	VVPRRFamide		

pQ; pyroglutamate. Modified from [19].

All neuropeptides are produced and secreted by highly regulated secretion pathways. In general, the precursors of neuropeptides are incorporated into the endoplasmic reticulum as a preprohormone where they are converted into prohormones. They are then transported to the Golgi apparatus where they undergo post-translational modifications such as endoproteolysis and *C*-terminal amidation before assuming their final active peptide forms. Cnidarian FLP cDNAs have been identified from several different animals. A cDNA from *Calliactis parasitica* contains 19 copies of Antho-RFamide, two copies of FQGRFamide, and one of YVPGRYamide [28]. In *A. elegantissima*, two cDNAs have been isolated. One has 13 copies of Antho-RFamide (Table 1) and nine other FLPs, whereas the other has 14 copies of Antho-RFamide (Table 1) and eight other FLPs [29]. In *Renilla kollikeri*, 36 copies of Antho-RFamide (Table 1) are present [30]. A cDNA from *Polyorchis penicillatus* has one copy of Pol-RFamide I (Table 1) and 11 copies of Pol-RFamide II (Table 1) along with another predicted FLP [31]. The Hydra-RFamides are encoded by three different preprohormones. Preprohormone-A contains all four Hydra-RFamides (Table 1) [23]. Preprohormone-B contains one copy of Hydra-RFamide I (Table 1), one copy of Hydra-Hydra RFamide II (Table 1), and two putative Hydra-RFamides [23]. Preprohormone-C contains one copy of Hydra-RFamide I (Table 1) and seven copies of putative neuropeptide sequences [23]. Collectively, cnidarian FLP cDNAs encoding the precursors yield many neuropeptides and structural diversity, indicating functional diversity.

### 2.2. GLWamides

GLWamides have characteristic structural features in their *N*- and *C*-terminal regions. For example, most of the peptides share a GLWamide motif at their *C*-termini (Table 2). In *Hydra*, seven GLWamide peptides [8,32] were isolated and found to have a proline residue at the second position (X-Pro) or at the second and third positions (X-Pro-Pro) of their *N*-terminal regions (Table 2). Metamorphosin A (MMA), which was isolated from the anthozoan *A. elegantissima* [9], has a pyroglutamyl residue at the *N*-terminus (Table 2). Both of these *N*-terminal structures confer resistance to aminopeptidase digestion [33].

**Table 2:** GLWamide-family peptides in Cnidaria.

Name	Peptide Sequence	Species	Reference
MMA	pQQPGLWamide	*Anthopleura elegantissima*	[34]
Hym-53	NPYPGLWamide	*Hydra magnipapillata*	[8,32]
Hym-54	GPMTGLWamide		
Hym-248	EPLPIGLWamide		
Hym-249	KPIPGLWamide		
Hym-331	GPPPGLWamide		
Hym-338	GPP^h^PGLWamide		
Hym-370	KPNAYKGKLPIGLWamide		
He-LWamide I	pQRPPGLWamide	*Hydractinia echinata*	[35]
He-LWamide II	KPPGLWamide		
Ae-LWamide I	pQQHGLWamide	*Actinia equine*	[35]
Ae-LWamide II	pQNPGLWamide		
Ae-LWamide III	pQPGLWamide		
Ae-LWamide IV	pQKAGLWamide		
Ae-LWamide V	pQLGLWamide		
Ae-LWamide VI	RSRIGLWamide		
Ae-MWamide	pQDLDIGMWamide		
MMA	pQQPGLWamide		
As-LWamide I	pQQAGLWamide	*Anemonia sulcata*	[35]
As-LWamide II	pQHPGLWamide		
As-IWamide	pQERIGIWamide		
Ae-LWamide II	pQNPGLWamide		
MMA	pQQPGLWamide		

pQ; pyroglutamate. ^h^P; hydroxyproline.

Cnidarian GLWamide cDNAs have also been identified in several different animals. Leviev *et al.* [34] cloned a cDNA encoding the preprohormone from *H. magnipapillata* containing 11 (eight different) immature peptide sequences. The cDNA encodes one copy each of Hym-53; NPYPGLWamide, Hym-54; GPMTGLWamide, and Hym-249; KPIPGLWamide, two copies of Hym-248; EPLPIGLWamide, and three copies of Hym-331; GPPPGLWamide along with three other predicted GLWamides (Table 2). One of the predicted peptides, termed Hydra-LWamide VIII, is likely to include GMWamide at the *C*-terminus [34]. In *Hydractinia echinata*, one cDNA encoding GLWamides was cloned [35]. The cDNA encodes one copy of He-LWamide I and 17 copies of He-LWamide II (Table 2). Compared with the preprohormone of GLWamides, two distinct cDNAs were cloned from the anthozoans, *Actinia equine* and *Anemonia sulcata* [35]. The cDNA from *Actinia* encodes one copy each of MMA, Ae-LWamide IV, Ae-LWamide V, Ae-LWamide VI, and Ae-MWamide, two copies each of Ae-LWamide I and Ae-LWamide III, and four copies of Ae-LWamide II (Table 2). On the other hand, the cDNA from *Anemonia* encodes one copy each of MMA, Ae-LWamide II, and As-IWamide, two copies of As-LWamide II, and four copies of As-LWamide I (Table 2) [35]. The original MMA is only contained in anthozoan preprohormones and not in hydrozoan preprohormones. Thus, MMA is a species-specific peptide. In addition, the peptide may be a prototype of the family with protection of the *N*-terminus by pyroglutamate [36]. Two further peptides that are possibly encoded in the preprohormone of *Actinia* and *Anemonia* are likely to be processed into -GMWamide (Ae-MWamide) and -GIWamide (As-IWamide) at the *C*-termini, respectively (Table 2). These two peptides do not belong to the GLWamide family, because replacement of Leu in GLWamide with Met or Ile results in complete or almost complete disappearance of contractile activity in the parietal muscle of *Anthopleura fuscoviridis* [37], suggesting that other novel neuropeptide families may exist.

### 2.3. Hym-355

The primary structure of Hym-355 is FPQSFLPRGamide (Table 3) [10]. Muneoka *et al.* [38] proposed to group peptides with a PRXamide sequence at their *C*-termini as PRXamide peptides. These are further divided into three sub-groups: (a) pheromone biosynthesis activating neuropeptides [39] and related peptides; (b) small cardioactive peptides [40,41]; and (c) antho-RPamide [33] and related peptides. Thus, PRXamide peptides are widely distributed in invertebrates. Hym-355; FPQSFLPRGamide shares some homology with members of the last group: LPPGPLPRPamide (Table 3), AAPLPRLamide from the echiuran, *Urechis unicinctus* [42], and QPPLPRYamide and pQPPLPRYamide from the snail, *Helix pomatia* [43].

**Table 3:** PRXamide peptides in Cnidaria.

Name	Peptide Sequence	Species	Reference
Hym-355	FPQSFLPRGamide	*Hydra magnipapillata*	[10]
Antho-RPamide	LPPGPLPRPamide	*Anthopleura elegantissma*	[33]

A persistent question regarding the comparative physiology of nervous systems is whether cnidarians contain the oxytocin-vasopressin superfamily of peptides, which are neurohypophysial hormones in vertebrates. In *Hydra*, vasopressin- and oxytocin-like immunoreactivity in the nervous system has been regarded as evidence for the presence of the oxytocin-vasopressin superfamily of peptides [44,45]. Morishita *et al.* [46] have purified two peptides, FPQSFLPRGamide (Hym-355) and SFLPRGamide, from *H. magnipapillata* using high performance liquid chromatography fractionation and immunological assays. They concluded that Hym-355; FPQSFLPRGamide and SFLPRGamide are the substances that account for the vasopressin-like immunoreactivity in the hydra nervous system. As Hym-355; FPGSFLPRGamide and vasopressin share the same sequence of the *C*-terminus (PRGamide) and both antibodies do not discriminate each other, Koizumi *et al.* [47] carried out immunohistochemical staining using anti-Hym-355 antibody and revealed that the antibody labeled the nerve rings in *Cladonema radiatum* and *Turritopsis nutricula* (order Anthomedusae, respectively). Whether Hym-355; FPQSFLPRGamide functions as a neurohypophysial hormone remains unclear.

## 3. Functional Diversity of Cnidarian Neuropeptides

### 3.1. Role of FLPs in Muscle Contraction, Feeding, Sensory Activity, Reproduction, Metamorphosis, and Larval Movement

Cnidarian FLPs mediate a variety of functions including control of muscle contractions, feeding, sensory activity, reproduction, metamorphosis, and larval movement. In the sea anemone *C. parasitica*, application of 0.1 to 1.0 μM Antho-RFamide causes an increase in tone, contraction amplitude, and frequency of slow muscle contraction [48]. The same peptide induces tonic contractions in the rachis and peduncle of the colony, and in the individual autozooid polyps of *R. kollikeri* with a threshold of 5 nM in summer colonies and 1 μM in winter colonies [49]. In *Hydra*, Hydra-RFamide III has a dose-dependent effect on the pumping activity of the peduncle [50]. Because the gastrovascular cavity not only digests food, but also delivers nutrients throughout the body, the authors suggested that the contractility of the peduncle is akin to cardiac activity in higher organisms.

A peptide-gated ion channel in snails is gated by FMRFamide [51,52]. Three ion channel subunits of the degenerin (DEG)/epithelial Na^+^ channel (ENaC) gene family have been cloned from *Hydra* and were named the *Hydra* Na^+^ channels (HyNaC) 2–4 [53]. A new subunit, termed HyNaC5, was cloned, and expression of this gene co-localizes with HyNaC2 and -3 at the base of the tentacles [54]. Co-expression of HyNaC5 with HyNaC2 and -3 in *Xenopus* oocytes greatly increases current amplitude after peptide stimulation and increases the affinity of the channel for Hydra-RFamide I and II [54]. A combination of HyNaC2/3/5 forms a peptide-gated ion channel of the DEG/ENaC gene family that contributes to fast neurotransmission in cnidarians. Analysis of a chimera between HyNaC and ENaC is intriguing regarding the evolutionary aspects of the ion channel. From analyses of HyNaCs, the authors speculated that release of Hydra-RFamide I and/or II leads to tentacle contractions, possibly when the animals are feeding [53,54]. Assmann and co-workers reported molecular cloning of seven more HyNaC subunits, HyNaC6 to HyNaC12, all of which are members of the DEG/ENaC gene family [55]. In *Xenopus* oocytes, these subunits assemble together with the four already known subunits into 13 different ion channels that are directly gated by Hydra-RFamide I and II with high affinity. Diminazene, an inhibitor of HyNaCs, delays tentacle movement in live *Hydra*. The authors showed that *Hydra* has a large variety of peptide-gated ion channels that are activated by a restricted number of FLPs [55]. Thus, *Hydra* may select FLPs for fast neuromuscular transmission. The possible function of Hydra-RFamide IV in *Hydra* remains unclear.

In addition to neurons, cnidarians have differentiated, highly specialized mechanoreceptor cells that play a pivotal role in the capture of prey and in defense [56]. These are phylum-specific stinging cells, named nematocytes. Ultrastructural studies showed the presence of two-cell and three-cell synaptic pathways in the tentacle epidermis of a sea anemone, including synaptic connections between nematocytes and surrounding neurons [57,58]. FLPs likely play a role in cnidarian sensory structures. The presence of immunoreactivity for FMRFamide and RFamide in the tentacles of animals from all four classes suggests that FLPs may be involved in the chemosensory regulation of cnidocyte discharge [59]. Also FMRFamide immunoreactivity has been seen in the epidermal sensory cells of the spot ocellus in *Aurelia* [60]. This neuronal control likely decreases the spontaneous firing activity of nematocytes.

FLPs are also involved in cnidarian reproduction, larval movement, and metamorphosis. Colonial octocorals such as *R. kollikeri* reproduce using the two-step process of spawning and exfoliation. During spawning, intact gamete follicles are released into the environment, and during exfoliation, the follicles rupture, freeing the gametes. Antho-RFamide, which is expressed in ciliated neurons within the follicle epithelia of *R. kollikeri*, induces the exfoliation of the follicle epithelium, releasing gametes into the surrounding medium [61]. Furthermore, the potency of the peptide is enhanced by light [61].

*H. echinata* is a colonial marine hydroid closely related to freshwater hydra. Fertilized eggs of this species undergo rapid cleavage divisions for about a day and develop into spindle-shaped planula larvae in about three days [62]. The planula larvae are able to migrate toward light [63], and they metamorphose into adult polyps when they receive appropriate environmental stimuli [64,65]. Hydra-RFamide I at 0.1 μM inhibits the migration of planula larvae, which shows modulatory action of phototaxis by inhibitory myomodulating activity [63]. Additionally, metamorphosis is also inhibited by the peptide, leading to the suggestion that the endogenous FLPs have a function in stabilizing the larval stage [66]. Thus, FLPs may play a role in regulation of movement of the planula prior to metamorphosis, possibly linking movement to chemotactic or phototactic processes [67]. Collectively, because sensory neurons expressing FLPs are present in the planula larvae, planula migration and metamorphosis may be regulated by the release of endogenous neuropeptides in response to environmental cues.

### 3.2. Role of GLWamides in Metamorphosis, Muscle Contraction, Planula Migration, Oocyte Maturation, and Spawning

Species of the genus *Hydractinia* are colonial and usually live on snail shells inhabited by hermit crabs. In their life cycle, only a planula larval stage exists with no medusa stage. Upon setting, the planula larvae undergo metamorphosis and develop into polyps after approximately one week [68]; MMA induces this metamorphosis [9]. This finding demonstrates that cnidarian neuropeptides function as neurohormones and control developmental processes in addition to playing roles as neurotransmitters and neuromodulators. *Hydra* GLWamide peptides also induce the metamorphosis of *H. serrata* planula larvae into polyps [8,32]. An *N*-terminal deletion series revealed that a common GLWamide sequence is necessary for the induction of metamorphosis in *Hydractinia*. Induction of metamorphosis is very specific for the GLWamide terminus and amidation is essential [69]. Furthermore, displacement of Gly of GLWamide with one of the other common amino acids (with the exception of Cys) resulted in a decrease or in disappearance of potency, and displacement of Leu or Trp of GLWamide with one of the other common amino acids (except Cys) resulted in complete or almost complete disappearance of potency in the muscle contraction of *A. fuscoviridis* [37]. However, the precise mechanisms of the actions of the GLWamide peptides in the induction of metamorphosis are not yet clearly understood. Interestingly, larvae can be induced to undergo metamorphosis in response to a chemical signal secreted by environmental bacteria [9]. This chemical signal is most likely received by the sensory neurons of the planula larvae, which then release endogenous GLWamide peptides that act on the surrounding epithelial cells, resulting in a change in the phenotype. Because hydra develop directly from embryos into adult polyps and have no intermediate larvae stage, the precise function of the GLWamide peptides in early embryogenesis in *Hydra* is still an open question.

Sexual reproduction in reef-building corals also involves motile planula larvae, which undergo complex metamorphosis after location of an appropriate substrate, founding a juvenile coral colony. In the coral genus *Acropora*, Iwao and co-workers found that Hym-248; EPLPIGLWamide induces metamorphosis of *Acropora* planula larvae into polyps at high rates (approximately 100%) and that *Acropora* planula respond to the peptide in a dose-dependent manner [70]. Interestingly, however, Hym-248; EPLPIGLWamide cannot induce metamorphosis in other coral genera [70,71]. Therefore, the specificity of ligand recognition by receptors appears to be dependent on the extent to which peptide(s) of particular structures are recognized in corals. In *Hydractinia*, the specificity is less stringent, and receptors can recognize any peptides belonging to the GLWamide family. Because Hym-248; EPLPIGLWamide is a surrogate ligand in *Acropora*, natural ligand(s) that are similar in structure to Hym-248; EPLPIGLWamide should be identified.

In *Hydra*, we found that all GLWamide peptides induce bud detachment from the parental polyp due to contraction of the sphincter muscle in the basal disk [8]. Tests of myoactivity typically employ epithelial *Hydra*, which are hydra with no nerve cells or any other cells that are derived from interstitial stem cells except for gland cells [72,73]. A similar effect was also observed in normal *Hydra* treated with the peptides. Unexpectedly, one of the *Hydra* GLWamide peptides, Hym-248; EPLPIGLWamide, not only induces bud detachment but also elongation of the body column [32]. *Hydra* muscle processes extending from the ectodermal and endodermal epithelial cells run perpendicular to each other. Hym-248; EPLPIGLWamide may have two types of receptors, one that is common to all GLWamide-family peptides and another that is specific to Hym-248; EPLPIGLWamide.

In *Anthopleura*, we also found that all GLWamide-family peptides induce contraction of the retractor muscle [32]. Immunohistochemical staining with an antibody specific for the GLWamide motif revealed intensely stained nerve cells in the retractor muscle of the sea anemone as well as in the nervous system of *Hydra* [32].

In *H. echinata*, migration of planula larvae is regulated by GLWamide and RFamide neuropeptides. One of the GLWamide family peptides, He-LWamide II (0.01 μM), stimulates migration primarily by lengthening the active periods [63]. As mentioned above, Hydra-RFamide I inhibits the migration of the planula larvae. Thus, GLWamides and FLPs work antagonistically to regulate migration in planula larvae of *H. echinata*.

**Figure 1:**
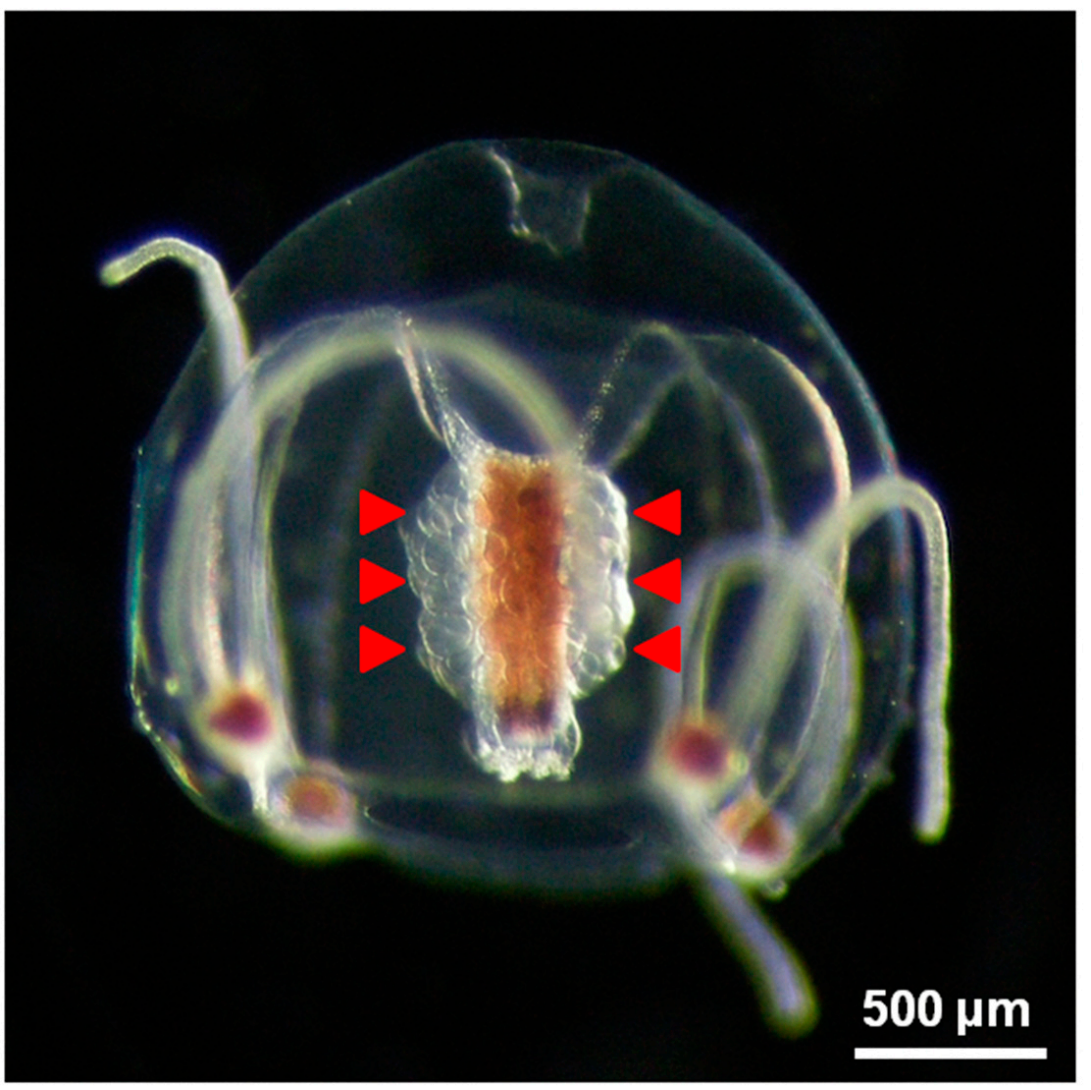
Morphology of an intact female *Cytaeis uchidae* medusa and released eggs. Fully grown oocytes in the ovary (red arrowheads) are visible around the manubrium.

Oocyte maturation and subsequent spawning in hydrozoan jellyfish are generally triggered by light-dark cycles in nature. In sexually mature female medusas of the hydrozoan jellyfish *Cytaeis uchidae* (Figure 1), a light period of 1 s is sufficient to trigger oocyte maturation and spawning in intact medusas or medusas without umbrellas, but the oocytes cannot resume meiosis unless they are kept inside the ovary for at least 4 min following light stimulation [74]. We revealed that the Hym-53; NPYPGLWamide-dependent period required for oocyte maturation and spawning is <2 min and that the onset time of spawning after neuropeptide treatment is comparable to that after light stimulation [75]. These observations suggest that neuropeptide(s) work as hormones to mediate the initial step that determines if oocytes undergo irreversible induction of meiotic maturation after light reception.

### 3.3. Roles of Hym-355 in Neuron Differentiation, Muscle Contraction, Oocyte Maturation, and Spawning

*Hydra* tissue is in a dynamic state and is constantly undergoing renewal due to continuous growth and differentiation of epithelial cells and interstitial stem cells. Nevertheless, neuronal density is maintained at a constant level. This neuronal homeostasis appears to be positively regulated by the neuropeptide Hym-355; FPQSFLPRGamide and negatively regulated by Pro-Trp (PW) peptide family members [8,10,76]. As PW peptides have a common sequence of Pro-Trp at their *C*-termini, we termed them the PW peptide family. The members are epitheliopeptides that are produced by epithelial cells [77].

Hym-355; FPQSFLPRGamide enhances neuronal differentiation at an early stage, whereas PW peptides, such as Hym-33H; AALPW, inhibit neuronal differentiation [10,76]. Furthermore, Hym-355; FPQSFLPRGamide and Hym-33H; AALPW show antagonistic properties, as treatment with one peptide nullifies the effect of treatment with the other peptide. Considering our data, these results suggest that a feedback model can explain the mechanism that regulates the homeostasis of neuronal differentiation in *Hydra* [10]. According to this model, Hym-355; FPQSFLPRGamide produced by neurons increases the rate of neuronal differentiation at an early stage in the pathway. On the other hand, to keep this effect in check, epithelial cells produce PW peptides that block neuronal differentiation. We propose the presence of a third factor that controls production and/or release of PW peptides from the epithelial cells and/or neurons. This tripartite mechanism presumably maintains a constant neuron density in *Hydra*. Hym-355; FPQSFLPRGamide is a neuropeptide that specifically enhances neuronal differentiation from interstitial stem cells in *Hydra* and also weakly promotes muscle contraction of the retractor muscle in the sea anemone *A. fuscoviridis* [10].

As mentioned above, Hym-53; NPYPGLWamide induces oocyte maturation and spawning. Hym-355; FPQSFLPRGamide also triggers these events, but the stimulatory effect is weaker than that of Hym-53; NPYPGLWamide. An antibody against Hym-355; FPQSFLPRGamide recognizes neurons located in the ovarian ectodermal epithelium [75]. Considering the effects of Hym-53; NPYPGLWamide and Hym-355; FPQSFLPRGamide on oocyte maturation and spawning in *Cytaeis*, we speculate that neurons that express neuropeptides may function downstream of light reception in the cnidaria. Future studies will be needed to isolate the endogenous neuropeptides that are responsible for the pathway, and will clarify the cell types that both release and respond to the molecules.

## 4. Conclusions

Peptides have long been recognized as important signaling molecules in the development and physiology of primitive metazoans such as cnidaria. In this review, we described 42 types of neuropeptides that have so far been identified in several species of cnidaria. Even in *Hydra*, 817 have been purified and 527 of these have been sequenced [24]. Thus, the study of neuropeptides in cnidaria is still in its infancy. We have initiated peptidomic analysis of *Hydra* combined with LC-MALDI-MS/MS and the *Hydra* expressed sequence tag database [12,77,78], the latter of which serves as a powerful tool to search for peptide receptors, which are generally GPCRs. Importantly, the approach we took here should be generally applicable to the study of signaling peptides in other organisms.

GLWamide-related peptides are present in higher metazoans [68], and GLWamide-like immunoreactivity has been observed in the cell bodies of neurons and in thin varicose fibers in some regions of the rat brain [79]. Novel neuropeptides are likely to provide a new and effective means to explore the mechanisms that underlie physiological and developmental processes in cnidarians and most likely will increase our understanding of peptide function in higher metazoans as well.

*Hydra* are often used as a model system for studying developmental mechanisms such as morphogenesis, patterning, and differentiation. Recently, Glauber *et al.* [80] initiated a *Hydra* head regeneration screen of a small molecule library and identified a novel small molecule, 6-(4-dimethylaminophenyl)-4-methylpyridin-2(1H)-one, which induces extra tentacles during regeneration. This is the first report of an unbiased small molecule screen for modulators of patterning in a whole-animal system. Retinoic acid (RA) is important for developmental processes in bilateria, and the RA receptor (RAX receptor; RXR) is involved in metamorphosis. Fuchs *et al.* [81] revealed that RA signaling is involved in the initiation of metamorphosis in response to a temperature shift in the moon jelly *Aurelia aurita*, in which the life cycle alternates between sessile asexual polyps and pelagic medusas. These findings underscore the importance of scyphozoan cnidarians in evolutionary studies and indicate that RA signaling is important for the life-cycle regulation machinery throughout the animal kingdom.
